# Infographics on risks associated with COVID-19 and the willingness to get the AstraZeneca vaccine: two randomized online experiments

**DOI:** 10.1186/s12889-024-18057-0

**Published:** 2024-02-20

**Authors:** Lisa Felgendreff, Regina Siegers, Leonie Otten, Cornelia Betsch

**Affiliations:** 1 Institute for Planetary Health Behaviour, University of Erfurt, Nordhäuser Str. 63, Erfurt, 99089 Germany; 2 Health Communication, BNITM Bernhard Nocht Institute for Tropical Medicine, Hamburg, Germany; 3 Data Literacy Project, Leibniz Institute for Educational Trajectories, Bamberg, Germany

**Keywords:** Vaccination, Confidence, Risk perception, Risk communication, Decision-making

## Abstract

**Background:**

Germans hesitated to get vaccinated with AstraZeneca in the COVID-19 pandemic after reports of blood clots.

**Methods:**

In two preregistered online experiments with stratified randomization (Study 1 *N* = 824, Study 2: *N* = 1,056), we tested whether providing evidence-based benefit-risk information reduces the perceived risk of the AstraZeneca vaccine and the perceived probability of blood clots due to the AstraZeneca vaccine and increases the vaccination intention. In Study 1, participants saw no infographic (control) or one of two infographics (low vs. high exposure risk varied by the underlying incidence rates). Study 2 additionally varied the infographic design displaying the risk information (presented as table, circle icons, or manikin-like icons).

**Results:**

The infographic decreased the risk perception of the vaccine compared to no infographic (Study 1: Cohens *d* = 0.31, 95% CI [0.14, 0.48]; Study 2: Cohens *d* = 0.34, 95% CI [0.06, 0.62]), but it did not influence the perceived probability of blood clots due to the AstraZeneca vaccine (Study 2: Cohens *d* = 0.05, 95% CI [-0.23, 0.33]). Also, the infographic design did not affect the perceived probability of blood clots (Study 2). The vaccination intention was not affected by viewing the infographic (Study 1: Cohens *d* = 0.04, 95% CI [-0.13, 0.21]; Study 2: Cohens *d* = 0.04, 95% CI [-0.24, 0.32]) nor the presented infection rate (Study 1: Cohens *d* = 0.07, 95% CI [-0.09, 0.24], Study 2: Cohens *d* = 0.01, 95% CI [-0.12, 0.15]) but by risk perceptions, sociodemographic characteristics, confidence in the AstraZeneca vaccine, and preference for alternative vaccines.

**Conclusions:**

The evidence-based benefit-risk information helped putting the risk of vaccinations into perspective. Nevertheless, objective risk information alone did not affect vaccination intention that was low due to the preexisting lacking vaccine confidence.

**Supplementary Information:**

The online version contains supplementary material available at 10.1186/s12889-024-18057-0.

## Background

Since the phase III trial, the manufacturer AstraZeneca has received negative media attention for its COVID-19 vaccine [1], especially due to concerns about suspected rare but severe side effects. During the investigation of these reports, several countries temporarily suspended use of AstraZeneca’s vaccine [2], and the perceived safety of this vaccine dropped as an immediate public reaction in some European countries [3, 4]. The European Medicines Agency concluded the investigation by stating that the “benefits still outweigh the risks despite possible link to rare blood clots with low blood platelets” [5]. Nevertheless, several countries changed their vaccination recommendations due to the possibility of specific types of blood clots [1], temporarily slowing down their vaccine rollout [6].

In Germany, changes in recommendations and suboptimal health communication created particularly challenging circumstances for the AstraZeneca vaccine. Early on, doubts existed about the vaccine’s efficacy in individuals that were older than 65 years as insufficient data were available to assess vaccine efficacy for this age group [7]. As a result, the German National Immunization Technical Advisory Group (STIKO) adapted their vaccination recommendations several times as knowledge about the vaccine’s effects increased (Additional file 1 – Supplement S1). The perceived safety of the AstraZeneca vaccine was already low since interim results of the phase III trials were published [8]. After pausing the AstraZeneca vaccine due to the safety reports, the German population’s confidence in its safety declined [8, 9]. At the same time, there was uncertainty about the likelihood of severe side effects due to this vaccine [9]. A month after these reports, safety perceptions had not recovered from their further drop [8]. The uptake of the AstraZeneca vaccine was low, leading health authorities to lift the prioritization rule (elderly first), making this vaccine the first that was available to the whole German adult population. However, despite generally high willingness to get vaccinated at the time [10], many doses remained unused. Other European countries, e.g., Denmark and France, also faced a lasting decrease in perceived safety of the AstraZeneca vaccine, while the perceived safety remained high throughout the debate in the United Kingdom [8]. The Danish Health Authority took the vaccine out of the campaign before Germany made the same decision [11]. Moreover, the temporal suspension of the AstraZeneca vaccine in several European countries and its associated news coverage had a short-term negative impact on the public’s general vaccination intention [12]. Other data from Germany [13] suggest that the long-term impact on general intentions to get vaccinated was low; it is, therefore, crucial to understand the mechanisms of risk perception and weighing of vaccine-specific and disease-associated risks in the decision process.

**Fig. 1:**
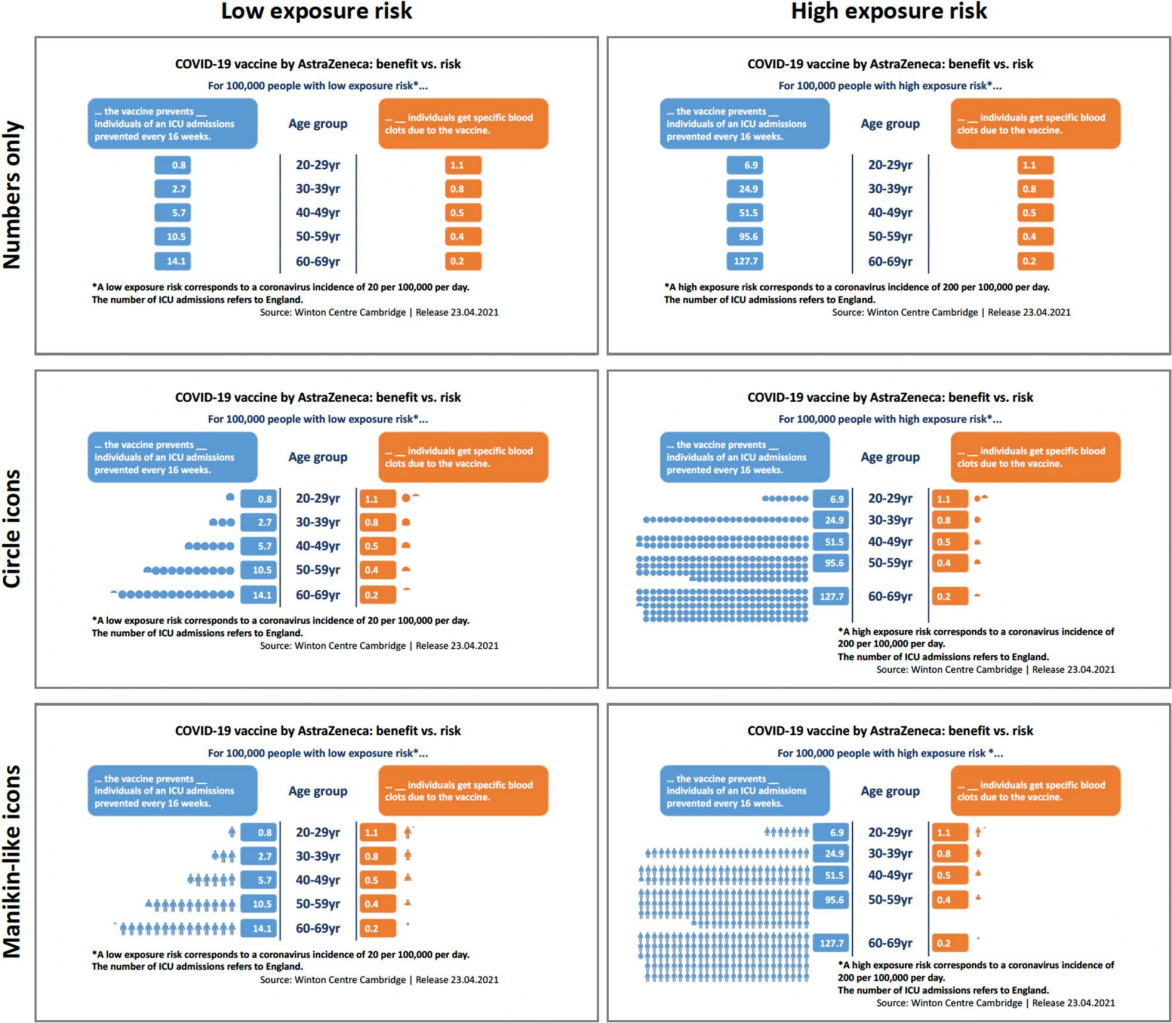
Overview of infographics used in Study 2 (English translation). Note. Study 1 used a slightly adapted version of the ‘Circle icons’ infographics in the middle panel

Moreover, it is important to understand the effect of evidence-based information about benefits and harms that could have a debiasing role when communicating about vaccination and disease risks. Transparent benefit-risk communication can promote informed decision-making and may help to balance the risks of the disease and the risks of the vaccine in the pandemic situation. In fact, while there were very rare cases of thrombosis associated with a reduction in the platelet count (thrombocytopenia) for those who received the AstraZeneca vaccine, there are also considerable risks from the disease, especially for older age groups who are more likely to suffer from more severe COVID-19 and when exposure risk is high. Thus, the vaccine may still have a better cost-benefit ratio than the prospect of getting infected, as the European Medicines Agency concluded on March 18, 2021 [5]. This line of thinking requires rational thinking and a rather “cold” comparison of risks. However, in a climate of high media attention and fears of side effects from a newly developed vaccine, this may be especially difficult for individuals who need to make a vaccination decision. Some media reports used infographics (e.g., like the one used in Studies 1 and 2, cf. Figure 1, panel ‘Circle icons’, put forward by the Winton Centre for Risk and Evidence Communication), which compared the risk of hospitalization due to a severe disease course and specific blood clots as vaccine adverse events per age group at different incidence rates. We tested, in two preregistered studies, whether providing such an infographic that puts risks into perspective can (1) reduce the perceived risk of developing blood clots and (2) increase the intention to vaccinate using the AstraZeneca vaccine.

### Study 1

Previous research has reported a consistent relation between perceiving vaccination risks and the intention to vaccinate—higher perceived risks of getting vaccinated are associated with lower vaccination intentions [14, 15]. In the case of the AstraZeneca vaccine, reports of blood clots occurring after receiving this vaccine were discussed extensively in the international media, highlighting the potential risk. These reports caused the public of several European countries to lower their safety perceptions of this vaccine [8]. To put the risk of vaccination in context, the Winton Centre for Risk and Evidence Communication provided an infographic using data from the United Kingdom comparing the risk of blood clots after vaccination and preventing the severe consequences of contracting COVID-19 [16]. This evidence-based information about the AstraZeneca vaccine may help lower the perceived risks of vaccination by balancing the disease risks and the risks of vaccinations during the pandemic and, thus, contribute to increase the vaccination intention. The *evidence-based information hypothesis* assumes that, compared to the control condition (no infographic), the intention to get vaccinated with AstraZeneca would be greater in conditions presenting evidence-based information about the comparative risk of severe COVID-19 vs. AstraZeneca’s risk of specific blood clots.

We presented infographics illustrating the benefit-risk profile of the AstraZeneca vaccine in either a low or high exposure risk scenario. The higher the exposure risk, the more instances of a severe COVID-19 outcome can be prevented by a vaccine, leading to greater benefits of the vaccine. Thus, in the high exposure risk scenario, the benefits of the AstraZeneca vaccine are more salient (i.e., the difference in prevented severe COVID-19 outcomes and caused instances of blood clots is greater than in the low exposure risk scenario). As greater perceived vaccine benefits are associated with a greater vaccination intention [17], the *pronounced benefit hypothesis* assumes that individuals who learn about the vaccine’s benefits within a high exposure risk scenario will have greater vaccination intentions than individuals who receive information within a low exposure risk scenario. We also explored the influence of risk perceptions, vaccination status, and socio-demographic variables on vaccination intention.

The presented materials provide evidence about the likelihood of a severe COVID-19 outcome vs. severe side effects. This information should affect the risk appraisal of getting vaccinated [17]. Therefore, the effect of the experimental material on the perceived risk of getting vaccinated was also explored.

## Method

### Participants and design

The preregistered experiment^1^ was part of the German cross-sectional survey series COVID-19 Snapshot Monitoring (COSMO), on April 20 and 21, 2021 [18]. The non-probabilistic sample was quota-representative for age × gender and federal state. Overall, *N* = 997 participants completed the survey. We excluded participants based on pre-registered criteria. Specifically, those aged below 20 and above 69 years were excluded, as the information material did not target these age groups, and individuals vaccinated twice or infected with COVID-19 were excluded (final sample *N* = 824). We used stratified randomization to balance the covariate age, since the risk information was presented by age (20–29, 30–39, 40–49, 50–59, and 60–69 years). Thus, participants were stratified according to their age group and randomly assigned to one of three experimental conditions: (1) no infographic (control), (2) infographic regarding low exposure risk, or (3) infographic regarding high exposure risk. The a priori sample size calculation for the *pronounced benefit hypothesis* (*f* = 0.15, α = 0.05, 1 – β = 0.95) resulted in *n* = 579 to detect a small effect in a one-factorial ANOVA comparing condition 2 and 3. An additional 290 participants were needed for the control group to test the *evidence-based information hypothesis*. Due to the exclusions, the final sample size slightly missed the target of *N* = 870 to detect small effects. The demographic characteristics did not vary between the experimental conditions (for sociodemographic details see Additional file 1: Supplement S3).

### Materials and measures

#### Infographic

The infographic displayed UK data [16] of the vaccine’s potential benefit and harm for every 100,000 people by age group. The potential benefits were prevented intensive care unit (ICU) admissions due to COVID-19 every 16 weeks. The low (high) exposure risk condition was based on an incidence of 2 (20) per 10,000 a day (compare Fig. 1, middle panel ‘Circle icons’ for slightly adapted infographics used in Study 1). The potential harms display the frequency of specific blood clots due to the vaccine, which were, of course, unrelated to the infection rates.

#### Dependent variables

Participants indicated their perceived risk of catching COVID-19 (‘How risky do you think COVID-19 is for you?’) and their perceived risk of getting vaccinated with the AstraZeneca vaccine (‘How risky do you think it is for yourself to be vaccinated against COVID-19 with the AstraZeneca vaccine?’) on a scale ranging from ‘*not at all risky*’ (1) to ‘*very risky*’ (7). The intention to get vaccinated with the AstraZeneca vaccine (‘How would you decide if you had the opportunity next week to be vaccinated against COVID-19 with AstraZeneca’s vaccine?’) was the main dependent variable and rated on a seven-point scale (1 = *definitely not vaccinate* to 7 = *definitely vaccinate*).

#### Procedure

Within the survey, participants saw a short introduction regarding the reports about specific blood clots, and those in the infographic condition received one of the two infographics. They viewed the information about the AstraZeneca vaccine, answered the risk perception questions, and rated their intention to get vaccinated as dependent variables on the same page.

#### Statistical analysis

To test the evidence-based information hypothesis, a one-factorial ANOVA was conducted with infographic as the independent variable and intention to get vaccinated as the dependent variable. The factor infographic contrasted both infographic conditions (grouped) with the control condition. For the pronounced benefit hypothesis, the factor infographic contrasted the conditions with low and high exposure risk in a one-factorial ANOVA. Cohens *d* was reported to facilitate effect size comparison for differences between two groups across dependent variables and studies. Bayes factors using the BIC approximation were reported when hypotheses tests suggested null effects [19]. Bayes Factors smaller than one indicate that the null hypothesis is favored over the alternative hypothesis. The closer the value is to zero, the stronger the evidence in favor of affirming the null hypothesis. All calculated Bayes factors were in favor of affirming the null hypothesis. Linear regression and a paired Welch’s *t*-test were used for explorative analyses concerning the intention to get vaccinated. The perceived risk of getting vaccinated was explored using the same procedure as described for vaccination intention. Analyses were performed with R version 4.1.0.

## Results

### Intention to get vaccinated with the AstraZeneca vaccine

**Fig. 2:**
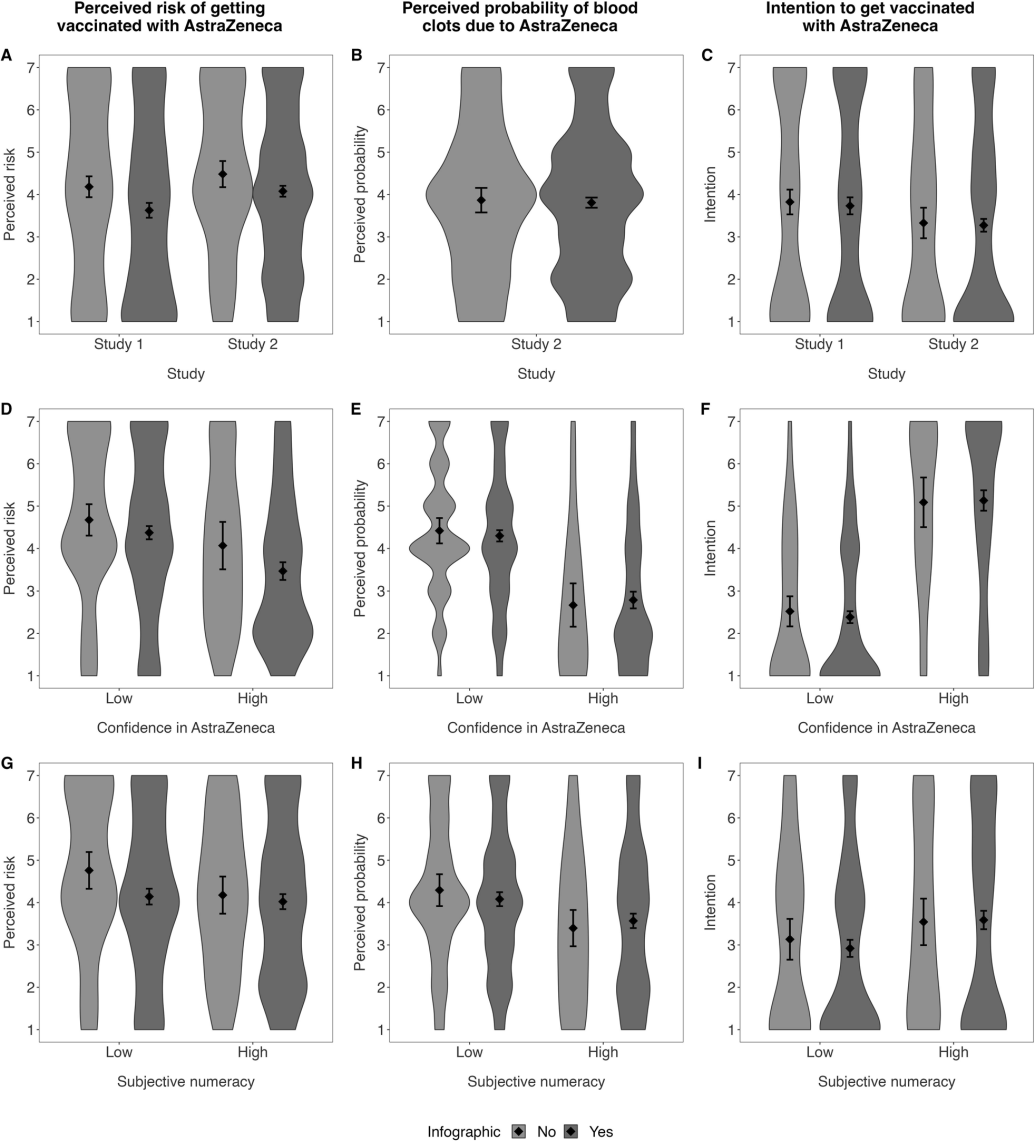
Distribution of dependent variables across different subgroups and studies. Note*.* The violin plots visualize the density distributions of the responses. The diamonds indicate group means and the whiskers represent the bootstrapped 95% confidence intervals. In both studies, presenting any infographic decreased the perceived risk of vaccination (**A**), while it neither influenced the perceived probability of getting blood clots due to the vaccine (**B**; only assessed in Study 2) nor the intention to get vaccinated with the AstraZeneca vaccine (**C**). In Study 2, greater confidence in vaccine safety was related to less perceived risk of vaccination (**D**) and less perceived probability of blood clots (**E**), as well as higher intentions to vaccinate (**F**). In Study 2, a higher subjective numeracy did not influence the perceived risk of vaccination (**G**), but was related to lower perceived probability of blood clots (**H**) and greater intentions to vaccinate (**I**). Numeracy did not generally affect the effect of the infographic’s impact

Figure 2 shows the main results. A one-factorial ANOVA showed that the mean vaccination intention did not differ between the control condition (*M* = 3.82, *SD* = 2.45, *n* = 273) and participants who received an infographic (*M* = 3.73, *SD* = 2.39, *n* = 551; *F*(1,822) = 0.26, *p* = 0.61, ${\eta }^{2}$ < 0.01, Cohens *d* = 0.04, 95% CI [-0.13, 0.21], BF = 0.04; Fig. 2C). Thus, the evidence-based information hypothesis was rejected. Contrary to our prediction in the pronounced benefit hypothesis, the intention to vaccinate did not differ when high exposure risk (*M* = 3.65, *SD* = 2.37, *n* = 280) or low exposure risk was displayed (*M* = 3.82, *SD* = 2.42, *n* = 271; *F*(1,549) = 0.75, *p* = 0.39, ${\eta }^{2}$ < 0.01, Cohens *d* = 0.07, 95% CI [-0.09, 0.24], BF = 0.06).

In a linear regression, we also explored the additional influence of risk perceptions, vaccination status, and socio-demographic variables on the intention to get vaccinated with AstraZeneca (Table 1). Perceiving a higher risk of COVID-19, a lower risk of the AstraZeneca vaccine, having already received a COVID-19 vaccine once, being older and male, and having a higher education led to higher vaccination intentions. However, the model only explained 19.6% of the variance.

We also explored whether the intention to get vaccinated with AstraZeneca was different from the general willingness to get vaccinated against COVID-19 (with no vaccine specified). This was the case, suggesting that AstraZeneca was a considerably less preferred vaccine (*M*_*AstraZeneca*_ = 3.76, *SD* = 2.41 vs. *M*_*in general*_ = 5.38, *SD* = 2.20, *n* = 824; paired Welch’s *t*-test, *t*(823) = -21.89, *p* < 0.001, Cohens *d* = -0.76, 95% CI [-0.84, -0.68]).

**Table 1:** Linear regression for intention to get vaccinated with the AstraZeneca vaccine (Study 1)

*Predictors*	**DV: Intention to get vaccinated with the AstraZeneca vaccine**				
	*B*	95% CI for *B*	*ß*	95% CI for *ß*	*p*
(Intercept)	1.62	0.89 – 2.34	-0.01	-0.16 – 0.14	** < 0.001**
Infographic with low exposure risk (control)	-0.01	-0.38 – 0.36	-0.00	-0.16 – 0.15	0.959
Infographic with high exposure risk (control)	-0.09	-0.45 – 0.28	-0.04	-0.19 – 0.12	0.646
Perceived risk of COVID-19	0.32	0.24 – 0.41	0.25	0.19 – 0.32	** < 0.001**
Perceived risk of getting vaccinated with the AstraZeneca vaccine	-0.09	-0.16 – -0.01	-0.08	-0.14 – -0.01	**0.019**
Age (years)	0.02	0.01 – 0.03	0.13	0.06 – 0.19	** < 0.001**
Gender (female)	-1.02	-1.32 – -0.72	-0.42	-0.55 – -0.30	** < 0.001**
University entrance qualification (no UEQ)	0.69	0.38 – 1.00	0.29	0.16 – 0.41	** < 0.001**
One shot of COVID-19 vaccine received (none)	1.14	0.74 – 1.54	0.47	0.31 – 0.64	** < 0.001**
N	824				
R^2^ / R^2^ adjusted	0.204 / 0.196				

The *p*-values in bold are statistically significant at *p* < 0.05

*DV* Dependent variable, *CI* Confidence interval

### Perceived risk of getting vaccinated with AstraZeneca

The explorative one-factorial ANOVA revealed a higher perceived risk of getting vaccinated in the control condition (*M* = 4.18, *SD* = 2.07, *n* = 273) compared to participants who received an infographic (*M* = 3.63, *SD* = 2.09, *n* = 551; *F*(1,822) = 12.94, *p* < 0.001, ${\eta }^{2}$ = 0.03, Cohens *d* = 0.31, 95% CI [0.14, 0.48]; Fig. 2A). Another ANOVA comparing both infographic conditions (low exposure risk: *M* = 3.70, *SD* = 2.06, *n* = 271; high exposure risk: *M* = 3.56, *SD* = 2.12, *n* = 280) showed no difference in the perceived risk of getting vaccinated depending on the exposure risk (*F*(1,549) = 0.59, *p* = 0.44, ${\eta }^{2}$ < 0.01, Cohens *d* = 0.7, 95% CI [-0.10, 0.23]).

## Discussion

Presenting an infographic about the benefits and risks of receiving the COVID-19 vaccine by AstraZeneca did not change the participants’ vaccination intention. Kerr et al. [20] provided information on vaccine efficacy and side effects in terms of evidence-based numbers, and this did not increase intentions to receive a COVID-19 vaccine. Instead, the identified predictors of this specific vaccination intention align with findings from many studies about COVID-19 vaccines in general: the perceived risk of COVID-19 increased the intention to get vaccinated against it while concerns about side effects of a (hypothetical) COVID-19 vaccine decreased the intention [21, 22]. In other studies, males and older participants also had stronger general vaccination intentions [21, 23]. Males and older persons are generally more at risk of severe illness or death due to COVID-19 [24]. In contrast, younger women may perceive reports about blood clots as being especially negative since these side effects were mistakenly compared to the thrombotic risk of oral contraceptives [25]. This potentially created the impression that young women were already at greater risk for blood clots. Concerning education, other studies also found that more educated participants had a higher intention to get vaccinated against COVID-19, in general [21, 26]. As with influenza vaccination [27], having already received one dose of a COVID-19 vaccine was a strong indicator of future intention to accept the AstraZeneca vaccine. Past behavior itself can form or reinforce perceptions that are congruent with respective future behavioral intentions [28]. Thus, participants who previously chose a COVID-19 vaccine were more likely to receive the AstraZeneca vaccine.

While the infographics did not affect the intention to get vaccinated, it lowered the perceived risk of getting vaccinated with AstraZeneca. Putting the risks of getting vaccinated into perspective changed how the vaccine itself was perceived. However, as we also found a large difference between the general intention to get vaccinated against COVID-19 vs. getting vaccinated with AstraZeneca, the difference in trust may be too large to be bridged by the provided risk-information alone. In Study 2, we sought to elaborate the finding for perceived risk associated with the AstraZeneca vaccine and explored additional predictors for vaccination intention.

### Study 2

The perception of risk is a central element in many health behavior models and is theorized as a function of the perceived probability and severity of a negative outcome [29]. Both components are associated with vaccine uptake [30]. The infographic presented in Study 1 explains the objective probability of a negative vaccine outcome; namely, it addresses the probability of blood clots due to the AstraZeneca vaccine. Thus, we specifically assessed this risk component in Study 2 to conceptually replicate the finding of Study 1 and further elaborate on the infographic’s influence on risk perception by focusing on the probability component of risk assessment. The *evidence-based probability hypothesis* expects that, compared to the control condition (no infographic), the perceived probability of blood clots due to the AstraZeneca vaccine decreases in conditions presenting evidence-based information about the comparative risk of severe COVID-19 vs. AstraZeneca’s risk of specific blood clots.

In Study 1, we did not observe a direct effect of the infographic on vaccination intention, but on the perceived risk of getting vaccinated measured with a compound measure (asking how risky people considered the vaccination and the disease to be). While previous research has shown that this measure correlates well with intentions [14, 15, 31], it is unclear whether the severity or the probability component of risk affected the intention to get vaccinated. In Study 2, we disentangled this and measured the components separately. The *risk-intention hypothesis* assumes that the intention to get vaccinated with AstraZeneca is higher in the case of (A) a greater perceived probability of becoming infected, (B) a greater perceived severity of becoming infected, (C) a lower perceived probability of blood clots, or (D) a lower perceived severity of blood clots. Furthermore, we explored the influence of socio-demographic variables, vaccine confidence, the tendency of benefit-risk weighing, and the preference of alternative COVID-19 vaccines on the intention to vaccinate.

Besides the presented content, the format of benefit-risk information can influence the understanding and risk perceptions as well. Visualizations such as icon arrays can elicit a more accurate risk perception than numeric information in text or Table [32]. Study 2 tested whether presenting the benefit-risk information either as purely numerical (table) or by illustrating the figures, leads to a different risk perception. The *illustration hypothesis* expects that, compared to presenting benefit-risk information numerically, the perceived probability of blood clots due to the AstraZeneca vaccine would be lower in conditions with icons illustrating the respective frequencies. There is still a debate about whether more concrete manikins or abstract icon types are best used to communicate risks and whether they differently affect risk perceptions. One study suggests that the correlation between actual and perceived risk is higher in more numerated and graphically literate persons viewing manikins instead of more abstract icons [33]. In practice, different icon types are used (e.g., manikins in Australia [34], circles in the UK [16], and squares by the European Medical Association [35]), probably due to personal preferences of graphical staff and not based on evidence. Therefore, we explored the effect of the infographic design on the perceived probabilities, also taking participants’ numeracy and graph literacy into account.

## Method

### Participants and design

The preregistered experiment was part of a survey conducted on May 18 and 22, 2021. The non-probabilistic sample was quota-representative for age × gender and federal state. Overall, *N* = 1,056 unvaccinated participants from Germany completed the survey. Participants aged below 20 and above 69 years old were excluded from the analysis (as the information material did not target these age groups), leaving *N* = 986 participants for analysis. In this study, we balanced randomization for the covariate age group (as in Study 1) and gender since the latter was also a crucial predictor of vaccination intention in Study 1. Participants were stratified according to their age and gender and randomly assigned to one condition of the 2 (infection rate: low vs. high) x 3 (infographic design: table, circles, manikins) + 1 (control) between-subjects design. The final sample exceeded the target sample size of *n* = 690 to detect small effects in a 2 × 3 ANOVA (*f* = 0.15, α = 0.05, 1 – β = 0.95) and additional *n* = 115 participants were needed for the control group. There were no differences in the demographic characteristics of the participants in the experimental conditions (for sociodemographic details, see Additional file 1: Supplement S4).

### Materials and measures

#### Infographic

The benefit-risk infographics on the AstraZeneca vaccine used in Study 1 were slightly adapted for Study 2. Some of the text was rephrased for better understanding. To test the effects of the infographic design, the figures were shown in three variants: (1) numbers only as a table, (2) numbers illustrated with circles, and (3) numbers illustrated with manikins (Fig. 1).

#### Understanding of AstraZeneca recommendation

Participants had to demonstrate their understanding of the current recommendation regarding the AstraZeneca vaccine in Germany by choosing the correct answer out of four options (correct answer: ‘The vaccine is officially recommended for people 60 years of age and older, but people younger than 60 can also be vaccinated with it.’).

#### Attitudes

Participants indicated their trust in the AstraZeneca vaccine’s safety (confidence) and their inclination to weigh the benefits and risks of the AstraZeneca vaccine to make the best possible decision (calculation) on a seven-point scale (1 = *do not agree at all* to 7 = *fully agree*) [36]. We also assessed the acceptance of the AstraZeneca vaccine when taking into account the availability of other manufacturer’s COVID-19 vaccines (preference of alternatives; e.g., ‘If I have a choice between different COVID-19 vaccines, I do not want to be vaccinated with AstraZeneca’s vaccine’) using the same scale (three items; *Cronbach’s alpha* = 0.76).

#### Dependent variables

Participants rated the risk of getting the AstraZeneca vaccine as in Study 1. They additionally rated their perceived probability of blood clotting due to the AstraZeneca vaccine (‘How likely do you think you are to have blood clots after vaccination with the AstraZeneca vaccine?’) and their perceived probability of becoming infected (‘How likely do you think you are to become infected with the coronavirus?’) on seven-point scales (1 = *extremely unlikely* to 7 = *extremely likely*). Participants stated their perceived severity of these outcomes (‘How severely would it affect your health if you had a blood clot after being vaccinated with the AstraZeneca vaccine?’; ‘How severe would an infection with the coronavirus be for yourself?’) on a scale ranging from ‘*completely harmles*s’ (1) to ‘*extremely dangerous*’ (7). Vaccination intention was assessed as in Study 1.

#### Cognitive skills

The Short Graph Literacy scale (four items; [37]) measured objective graph literacy and the Subjective Numeracy Scale (three items; [38]) measured subjective numeracy. For exploratory analyses, graph literacy and subjective numeracy were divided into two groups (low and high) by a median split.

### Procedure

Study 2 was part of a survey comprising two experiments where the other experiment always preceded Study 2. The first section examined the influence of social media posts on vaccination willingness.^2^ In the second section, Study 2 started with a short introduction on the reports about specific blood clots and the official recommendations for the AstraZeneca vaccine. Participants had to pass a comprehension check on the vaccination recommendation to proceed with the questionnaire. The correct answer was presented after two failed attempts, and participants had to select the correct option to continue with the next question. They then answered the measures in the order reported above.

### Statistical analysis

The passing rates across the experimental conditions were compared using the *Χ*^*2*^-test. A Welch’s *t*-test for independent groups compared the perceived probability of blood clots due to the AstraZeneca vaccine in the control group to the conditions receiving any infographic (evidence-based probability hypothesis). To test the illustration hypothesis, we conducted a 2 (exposure risk) x 2 (infographic design: table vs. any icons) ANOVA. The results were elaborated by exploring the role of subjective numeracy and graph literacy in two 2 × 3 × 2 ANOVAs. Cohens *d* was reported for differences between two groups to facilitate effect size comparisons across both studies. The risk-intention hypothesis was tested by using linear regression with two steps. The first step included the variables stated in the hypothesis, and the second included socio-demographic and vaccine-related variables for explorative purposes. As in Study 1, Bayes factors using the BIC approximation [19] were reported if hypothesis testing results favored the null hypothesis and supported this interpretation. Analyses were conducted in R version 4.1.0.

## Results

Figure 2 shows the distribution of the dependent variables as a function of the experimental conditions, confidence in the AstraZeneca vaccine, and subjective numeracy (see Additional file 1: Supplement S5 for detailed statistical analyses).

### Understanding of the AstraZeneca recommendation

After reading the introduction about current regulations for the AstraZeneca vaccine, 79.61% of the participants gave the correct answer about the official vaccination recommendation on the first attempt. The proportion of those who gave the correct answer did not differ across the experimental conditions (*Χ*^*2*^(6) = 4.85, *p* = 0.56). Rerunning the following analyses excluding participants who failed the question on the first attempt yielded similar results (see Additional file 1: Supplement S6).

### Perceived probability of blood clots due to the AstraZeneca vaccine

A Welch’s *t*-test for independent groups was conducted to test the evidence-based probability hypothesis. As can be seen in Fig. 2B, and contrary to our expectations, participants in the control condition without an infographic did not perceive a higher probability of blood clots as a result of the AstraZeneca vaccine (*M* = 3.87, *SD* = 1.75, *n* = 143) compared to participants seeing evidence-based information (*M* = 3.81, *SD* = 1.78, *n* = 843; *t*(194.88) = 0.37, *p* = 0.71, Cohens *d* = 0.05, 95% CI [-0.23, 0.33], BF = 0.03).

A 2 (exposure risk) x 2 (infographic design: table vs. any icons) ANOVA with infographic design grouped to contrast purely numerical with icon-illustrated information yielded no significant main effects or interaction (all *F*s < 0.5). Also, contrary to our expectations, the perceived probability of blood clots due to the AstraZeneca vaccine was not lower in conditions presenting benefit-risk information numerically (*M* = 3.84, *SD* = 1.82, *n* = 283) compared to conditions with icons illustrating the respective frequencies (*M* = 3.79, *SD* = 1.76, *n* = 560; *F*(1,837) = 0.16, *p* = 0.69, ${\eta }_{p}^{2}$ < 0.01, Cohens *d* = 0.03, 95% CI [-0.13, 0.20], BF = 0.04). Thus, the illustration hypothesis was rejected.

We then explored the effect of infographic design on the perceived probability of blood clots due to the AstraZeneca vaccine considering both numeracy and graph literacy. First, a 2 (exposure risk) x 3 (infographic design) x 2 (subjective numeracy) ANOVA was conducted. Participants with lower subjective numeracy (*M* = 4.11, *SD* = 1.67, *n* = 471) perceived a higher risk of blood clots than those with a higher subjective numeracy (*M* = 3.55, *SD* = 1.83, *n* = 515; *F*(1,831) = 17.47, *p* < 0.001, ${\eta }_{p}^{2}$ = 0.02, Cohens *d* = 0.33, 95% CI [0.20, 0.45]). There was no interaction effect of the infographic design and subjective numeracy (*F*(2,831) = 0.17, *p* = 0.85, ${\eta }_{p}^{2}$ < 0.01). None of the other effects reached statistical significance (all other *F*s < 3.8). Second, a 2 (exposure risk) x 3 (infographic design) x 2 (graph literacy) ANOVA showed the same pattern of results. Only the main effect of graph literacy was significant (*F*(1,831) = 7.41, *p* = 0.007, ${\eta }_{p}^{2}$ < 0.01, Cohens *d* = 0.17, 95% CI [0.04, 0.29]) with less graphically literate participants (*M* = 3.98, *SD* = 1.73, *n* = 434) perceiving a higher risk of blood clots than those who were more graphically literate (*M* = 3.69, *SD* = 1.80, *n* = 552). All other effects, including the interaction effect of infographic design and graph literacy were not significant (all other *F*s < 1.6).

### Intention to get vaccinated with the AstraZeneca vaccine

The risk-intention hypothesis was tested by conducting a linear regression. As predicted, the intention to get vaccinated with AstraZeneca was higher in the case of (A) greater perceived probability of becoming infected, (B) greater perceived severity of becoming infected, (C) lower perceived probability of blood clots, and (D) lower perceived severity of blood clots (Table 2, Step 1). The model explained 39.0% of the variance in the vaccination intention. For exploratory proposes, we added socio-demographic variables, vaccine confidence, calculation, and the preference of alternative COVID-19 vaccines as predictors to regression in a second step. The explained variance of the vaccination intention increased to 61.5%. Being older, male, having a school education with university entrance qualification, being confident in the AstraZeneca vaccine, and not preferring an alternative COVID-19 vaccine increased the intention to get vaccinated with the AstraZeneca vaccine. The desire to weigh the benefits and risks of the AstraZeneca vaccine (calculation) did not systematically increase or decrease vaccination intention (Table 2, Step 2).

**Table 2:** Linear regression for intention to get vaccinated with the AstraZeneca vaccine (Study 2)

*Predictors*	**DV: Intention to get vaccinated with the AstraZeneca vaccine**									
	*Step 1*					*Step 2*				
	*B*	95% CI for *B*	*ß*	95% CI for *ß*	*p*	*B*	95% CI for *B*	*ß*	95% CI for *ß*	*p*
(Intercept)	5.62	5.12 – 6.12	-0.00	-0.05 – 0.05	** < 0.001**	2.83	2.21 – 3.45	-0.00	-0.07 – 0.07	** < 0.001**
Perceived probability of becoming infected with COVID-19	0.19	0.11 – 0.27	0.13	0.07 – 0.19	** < 0.001**	0.15	0.08 – 0.22	0.10	0.06 – 0.15	** < 0.001**
Perceived severity of becoming infected with COVID-19	0.27	0.19 – 0.35	0.19	0.14 – 0.25	** < 0.001**	0.11	0.04 – 0.18	0.08	0.03 – 0.13	**0.002**
Perceived probability of blood clots due to the AZ vaccine	-0.53	-0.60 – -0.46	-0.42	-0.48 – -0.37	** < 0.001**	-0.20	-0.26 – -0.14	-0.16	-0.21 – -0.12	** < 0.001**
Perceived severity of blood clots due to the AZ vaccine	-0.38	-0.46 – -0.30	-0.26	-0.31 – -0.21	** < 0.001**	-0.14	-0.21 – -0.07	-0.09	-0.14 – -0.05	** < 0.001**
Age (years)						0.01	0.01 – 0.02	0.09	0.05 – 0.14	** < 0.001**
Gender (female)						-0.28	-0.46 – -0.11	-0.13	-0.21 – -0.05	**0.002**
University entrance qualification (no UEQ)						0.27	0.08 – 0.45	0.12	0.04 – 0.20	**0.005**
Confidence in AZ						0.52	0.46 – 0.58	0.46	0.41 – 0.51	** < 0.001**
Calculation regarding AZ						-0.02	-0.08 – 0.03	-0.02	-0.06 – 0.03	0.454
Preference of alternatives to AZ						-0.28	-0.35 – -0.21	-0.20	-0.25 – -0.15	** < 0.001**
N	986					986				
R^2^ / R^2^ adjusted	0.392 / 0.390					0.619 / 0.615				

Exploratory variables were added in step 2. The *p*-values in bold are statistically significant at *p* < 0.05

*AZ* AstraZeneca vaccine, *UEQ* University entrance qualification, *DV* dependent variable, CI confidence interval

## Discussion

In Study 2, we aimed to elaborate on how the benefit-risk infographic decreased the perceived risk of getting vaccinated with the AstraZeneca vaccine by focusing on the probability component of the risk assessment. However, although presenting the infographic led to lower perceived risk of being vaccinated with the AstraZeneca vaccine in Study 2 (replicating the effect found in Study 1), it did not influence the perceived probability of blood clots due to the AstraZeneca vaccine. Thus, the adapted overall risk of vaccination judgement potentially reflected the infographic’s main function, namely, to put the risk of the vaccine in relation to its benefit. Providing such a balanced information format for the AstraZeneca vaccine seems critical since the judgment of benefits and risks are often inversely related because of biases in information processing [39]. This phenomenon has already been observed for vaccines such that more beneficial vaccines are perceived as having less risk and vice versa [39, 40]. Research suggests as underlying mechanism that the overall affective judgment systematically influences the evaluation of benefits and risks in people’s minds [39, 41]. Therefore, presenting the ICU admissions that were prevented due to the AstraZeneca vaccine makes its positive impact obvious and we cautiously interpret that it is also incorporated in the overall judgment about the vaccine.

Visualizing the information with icons (circles or manikins) instead of a table did not change the perceived probability of blood clots or COVID-19 infection when compared to seeing numbers only. Thus, these results are not in line with previous findings that showed that illustrating numbers with icons facilitated an appropriate risk perception in general and especially for individuals with lower numeracy and graphical literacy [42, 43]. However, the lacking effect of illustrating the numbers with icons could be due to the infographic’s design. The icons only illustrated the numerators, but not the denominator, thereby reinforcing the focus on comparing frequencies between age groups and between vaccine benefit and risk. Not visualizing the denominator with icons resulted in failing to highlight the part-to-whole relationship [44] and thus, the benefit of a graphic illustration for lower numerate participants may have diminished.

Additionally, the choice of icon type seems to not be a matter of concern for the tested iconicity levels; the perceived risk did not vary between the conditions with more concrete manikin and more abstract circle icons, even when considering numeracy and graph literacy. Instead, the cognitive skills directly influenced the risk perception, regardless of the presented infographic. Participants with lower subjective numeracy and a lower graph literacy perceived a higher probability of getting blood clots from the AstraZeneca vaccine. This is in line with previous research results showing that experiencing difficulties in interpreting numerical and graphical information often leads to increased risk perception [37, 45]. The pure presence of the possibility of adverse events may have been sufficient to elicit a diffuse feeling of risk in low-literate participants.

Since the low vaccination intention was not based on lacking benefit-risk information in Study 1, Study 2 explored the explanatory value of the psychological antecedence factors of vaccine confidence, preference of alternative COVID-19 vaccine and the tendency of benefit-risk weighing. As with confidence in COVID-19 vaccines in general, confidence in the AstraZeneca vaccine was also an important predictor of vaccination intentions [46]. Due to the low confidence levels in the AstraZeneca vaccine in Germany [47], an essential driver of the vaccination intention for the AstraZeneca vaccine was missing. Furthermore, the news coverage on the COVID-19 pandemic put the German public into the position to compare vaccines of different manufacturers and form a preference. Several studies show that many citizens prefer vaccines originating from their own country [48–50]. Individuals with a strong preference for a certain vaccine have a high vaccination intention when offered their preferred vaccine. However, when offered an alternative vaccine, their intention is usually lower than their general vaccination intention [51]. This phenomenon described by Sprengholz et al. [51] could possibly explain why the availability of alternative vaccines lowers the intention to get vaccinated with the AstraZeneca vaccine in Study 2. However, having a strong need to weigh the benefits and risks of the AstraZeneca vaccine (calculation) did not explain the vaccination intention. Thus, the inclination to consider benefits and risks of the AstraZeneca vaccine in the vaccination decision could lead to both a low or high intention to get vaccinated.

## General discussion

Study 1 showed that the AstraZeneca vaccine was, indeed, not very attractive to Germans, as the intention to get this specific vaccine was lower than the general intention to get vaccinated against COVID-19. Providing an infographic with evidence-based benefit-risk information did not increase vaccination intention, but still lowered the perceived risk related to getting vaccinated with the AstraZeneca vaccine. Previous studies on the effects of COVID-19 vaccine information showed that balanced communication formats can foster an understanding of vaccine benefits and risks [20, 52] even though they might not increase vaccination intention [20]. In particular, skeptical and undecided individuals were found to adjust their evaluation of the vaccine’s benefit-risk ratio if they understood the information [52]. Furthermore, strongly hesitant individuals found the information about the personal benefit of vaccination appealing [53]. The results for the perceived risk of getting vaccinated with the AstraZeneca vaccine underline the importance of a combined communication about risks of vaccinations and their benefits to enable an informed overall vaccine judgment.

Deliberations and feelings are essential determinants of vaccination intention, and despite great efforts of researchers and practitioners, there are no effective interventions for people with low confidence levels [54]. Given the Germans’ low preexisting confidence in the AstraZeneca vaccine, it is not surprising that the tested, evidence-based benefit-risk information had no impact on the vaccination intention. Even the higher figures of prevented ICU admissions in the high exposure risk condition could not facilitate a more positive vaccine judgment, let alone increase vaccination intention. The infographic used in this study educated the participants on vaccine safety through objective, deliberate risk information. Thus, it may only enable highly numerate people to derive affective meaning from this numeric information [55]. Further studies should test whether communication formats that also address affective risk components are more effective in facilitating an adequate benefit-risk assessment for vaccines that health authorities determined are save.

An important limitation of these studies is that ICU admissions referred to the United Kingdom. The German participants might have preferred to see the figures for ICU admissions in Germany. However, this information was not available for Germany or the European Union at the time of Study 1. Furthermore, in Study 1, the intention to get vaccinated with the AstraZeneca vaccine was higher for previously vaccinated individuals. The participants did not state the vaccine type they originally received. Thus, we do not know how the vaccine type influenced the relationship between vaccination intention and past behavior. Finally, vaccine confidence is highly country dependent. On the one hand, the public of several European countries judged the AstraZeneca vaccine as being unsafe even four months after the initial reports of blood clots [47]. On the other hand, in the United Kingdom, where the vaccine was developed, the reports hardly affected the perceived safety of the AstraZeneca vaccine. Many differences between the countries exist, not only how hard COVID-19 hit the countries, but also the way that adverse events were communicated in the countries (the graph originates from the United Kingdom); further, AstraZeneca was developed in the United Kingdom, which may also lead to a more positive evaluation or higher trust in the vaccine. Thus, generalizing the results to other countries seems difficult; the micro effects that the infographics had on risk perceptions should be replicated independently from these factors.

## Conclusions

In sum, the presented studies on the backdrop of the German AstraZeneca vaccination crisis highlight the importance of responsible and transparent reporting of vaccination risks and benefits. Once confidence is down, it is hard to regain. In this case, even evidence-based benefit-risk communication may not be sufficient to enable an adequate overall vaccine judgment.

### Supplementary Information

**Additional file 1.** Supplementary information and analyses.

## Data Availability

The preregistration, materials, data sets, analysis scripts, and supplements used during the current study are available in the Open Science Framework repository 10.17605/OSF.IO/82KP6.

## Footnotes

1 Studies 1 and 2 were preregistered (anonymized version of the pre-registrations: https://aspredicted.org/w5uz7.pdf, https://aspredicted.org/z83iw.pdf). Reported hypotheses were performed according to the preregistered analysis plans. See Additional file 1: Supplement S2 for not reported preregistered hypotheses of Study 2.

2 Explorative t-tests comparing the means of Study 2’s dependent variables for those two conditions did not indicate any difference in the group means (see Additional file 1: Supplement S7). Since Studies 1 and 2 yield similar effects, the stimuli of the preceding experiment seemed not to have influenced Study 2’s dependent variables. Overall, the setting of the survey seems to yield low potential to have influenced the results of Study 2.
