# Supplementary material for: Infographics on risks associated with COVID-19 and the willingness to get the AstraZeneca vaccine: two randomized online experiments
Source: BMC Public Health. 2024 Feb 20;24:529. doi: 10.1186/s12889-024-18057-0 (PMC10880230; doi:10.1186/s12889-024-18057-0)
Supplement: Supplementary file 1 — Additional file 1. Supplementary information and analyses. [file 12889_2024_18057_MOESM1_ESM.docx]

# **Supplement**

## Supplement S1

**Figure 1.**


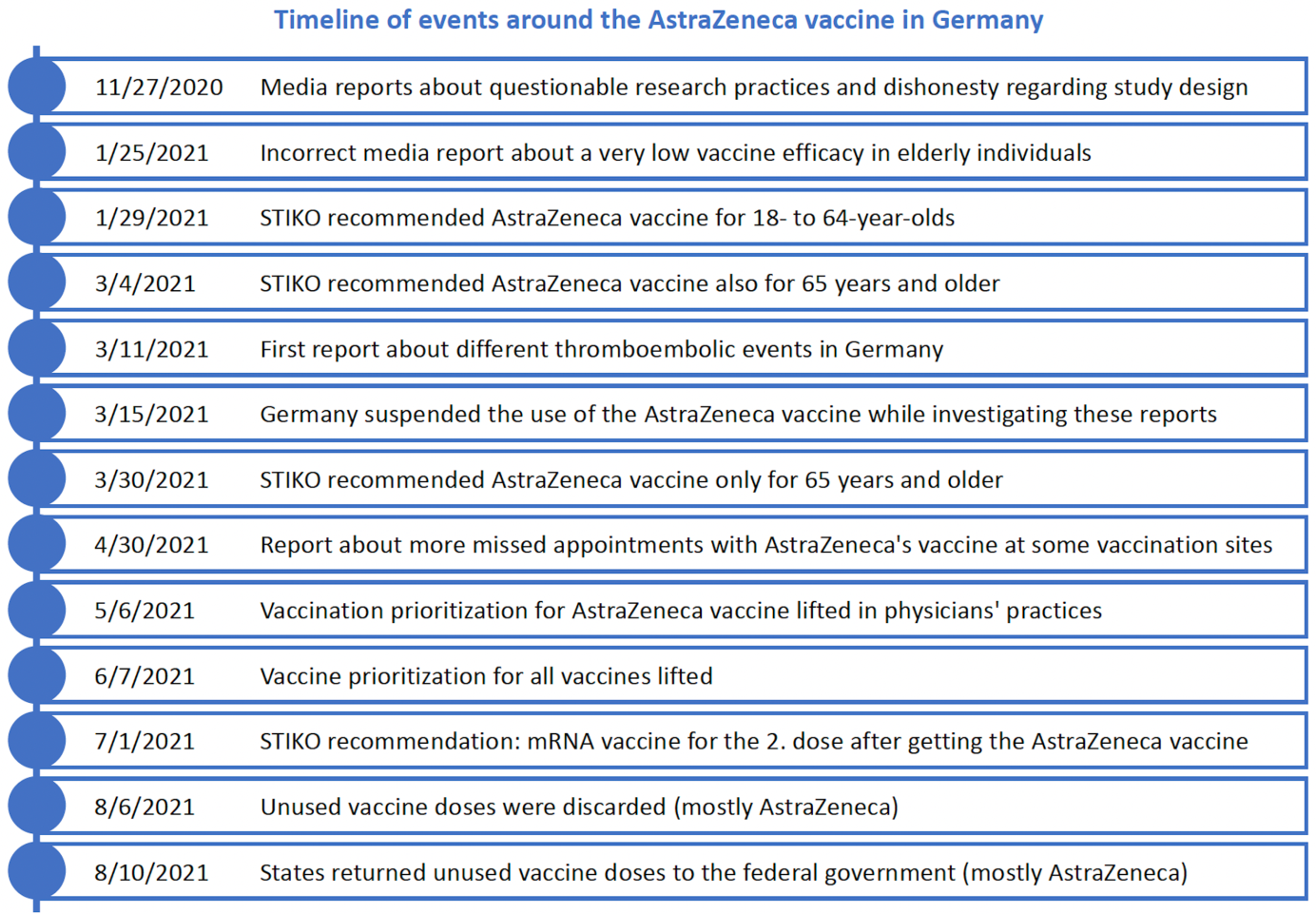
*Timeline of events around the AstraZeneca vaccine in Germany.*

*Note.* STIKO (Ständige Impfkommission) is the German National Immunization Technical Advisory Group. After March 30, 2021, adults younger than 65 could still receive the AstraZeneca vaccine after detailed medical consultation, but it was no longer recommended.

## Supplement S2

**Preregistered hypotheses not reported in the paper**

Two preregistered hypotheses were not reported in the paper due to a mismatch between them and the information provided in the infographic:

- **Hypothesis 2 (AsPredicted.org):** Compared to the illustration of a low incidence, the perceived probability of becoming infected with the coronavirus increases in the condition with an infographic illustrating a high incidence.
- **Hypothesis 4 (AsPredicted.org):** When the risk of an ICU admission is depicted with icons (instead of numbers in a table), the difference in the perceived probability of becoming infected with the coronavirus will be more pronounced when comparing low and high incidence.

**Results.** A 2 (infection risk) x 3 (infographic) ANOVA with perceived probability of becoming infected with COVID-19 as the dependent variable and the factor infographic grouped as a priori defined (table vs. any icons) was conducted to test Hypotheses 2 and 4. No main effects nor interaction effects reached statistical significance (all *F*s < 3.3). Hypotheses 2 and 4 were rejected. The perceived probability of becoming infected with the coronavirus was not higher when participants viewed an infographic illustrating vaccine benefits in a high incidence (*M* = 3.51, *SD* = 1.51, *n* = 423) instead of a low incidence scenario (*M* = 3.70, *SD* = 1.55, *n* = 420, BF = 0.15; H2). Also, the level of risk exposure did not interact with the design of the infographic (BF = 0.06; H4).

**Discussion.** The perceived probability of becoming infected with the coronavirus (‘How likely do you think you are to become infected with coronavirus?’, 1 = extremely unlikely, 7 = extremely likely) was not influenced by the infection rate or the type of infographic viewed. One reason for the lacking influence could be the mismatch between these hypotheses and the information provided in the infographic. In the presented infographic, the vaccine benefit is illustrated by the number of ICU admissions and not by the number of infections. We suspected that although the media reported extensively on the 7-day-incidence rate of new infections and the shortage of ICU beds, people probably did not know their age-specific risk for a severe disease course at different levels of exposure risks. Regardless of age, the higher the infection rates, the higher the ICU admissions. Presumably, participants did not infer the infection probability from the number of ICU admissions. Furthermore, the actual 7-day-incidence rate of new infections in Germany might have a greater weight on the perceived probability than the exposure scenario described.

## Supplement S3

**Table S.1**

*Sociodemographic details of Study 1 (N = 824)*

| Variable | Distribution parameters | | Comparison across experimental conditions |
| --- | --- | --- | --- |
| **Age** | *M (SD)* | 45.73 (14.60) | *F*(2,821) = 0.146, *p* = .86 |
| **Gender** |  |  | *Χ^2^*(2) = 1.578, *p* = 0.45 |
| Male | *n* (%) | 397 (48.18) |  |
| Female | *n* (%) | 427 (51.82) |  |
| **Education** |  |  | *Χ^2^*(2) = 0.355, *p* = 0.84 |
| Without university entrance qualification^a^ | *n* (%) | 361 (43.81) |  |
| University entrance qualification | *n* (%) | 463 (56.19) |  |
| **Vaccination status** |  |  | *Χ^2^*(2) = 5.651, *p* = 0.06 |
| None | *n* (%) | 679 (82.40) |  |
| One shot | *n* (%) | 145 (17.60) |  |
| Two shots^b^ | *n* (%) | - |  |
| **Infection status** |  |  | N/A |
| Infected | *n* (%) | 824 (100.00) |  |
| Not infected^b^ | *n* (%) | - |  |
| **State** |  |  | N/A |
| Baden-Württemberg | *n* (%) | 102 (12.38) |  |
| Bavaria | *n* (%) | 131 (15.90) |  |
| Berlin | *n* (%) | 30 (3.64) |  |
| Brandenburg | *n* (%) | 26 (3.16) |  |
| Bremen | *n* (%) | 7 (0.85) |  |
| Hamburg | *n* (%) | 19 (2.31) |  |
| Hesse | *n* (%) | 56 (6.80) |  |
| Mecklenburg-Vorpommern | *n* (%) | 19 (2.31) |  |
| Lower Saxony | *n* (%) | 78 (9.47) |  |
| North Rhine-Westphalia | *n* (%) | 182 (22.09) |  |
| Rhineland-Palatinate | *n* (%) | 40 (4.85) |  |
| Saarland | *n* (%) | 10 (1.21) |  |
| Saxony | *n* (%) | 42 (5.10) |  |
| Saxony-Anhalt | *n* (%) | 27 (3.28) |  |
| Schleswig-Holstein | *n* (%) | 30 (3.64) |  |
| Thuringia | *n* (%) | 25 (3.03) |  |

*Note.* A one-factorial ANOVA (metric variable level) or *Χ^2^*-test (nominal variable level) was conducted to test for differences between the three conditions. N/A implies that comparison was not possible (only one group) or reasonable (this is the case for the variable state). ^a^ The response options “Up to 9 years of school education” and “At least 10 years (without university entrance qualification)” were summarized to “Without university entrance qualification” for statistical analysis. ^b^ Participants indicating this response option were excluded from the final sample.

## Supplement S4

**Table S.2**

*Sociodemographic details of Study 2 (N = 986)*

| Variable | Distribution parameters | | Comparison across experimental conditions |
| --- | --- | --- | --- |
| **Age** | *M (SD)* | 44.81 (14.24) | *F*(6,979) = 0.195, *p* = 0.98 |
| **Gender** |  |  | *Χ^2^*(6) = 0.293, *p* = 1.00 |
| Male | *n* (%) | 496 (50.30) |  |
| Female | *n* (%) | 490 (49.70) |  |
| **Education** |  |  | *Χ^2^*(6) = 1.104, *p* = 0.98 |
| Without university entrance qualification^a^ | *n* (%) | 452 (45.84) |  |
| University entrance qualification | *n* (%) | 534 (54.16) |  |
| **Vaccination status** |  |  | N/A |
| None | *n* (%) | 986 (100.00) |  |
| One shot^b^ | *n* (%) | - |  |
| Two shots^b^ | *n* (%) | - |  |
| **Infection status** |  |  | *Χ^2^*(6) = 2.401, *p* = 0.90 |
| Infected | *n* (%) | 60 (6.09) |  |
| Not infected | *n* (%) | 926 (93.91) |  |
| **State** |  |  | N/A |
| Baden-Württemberg | *n* (%) | 128 (12.98) |  |
| Bavaria | *n* (%) | 152 (15.42) |  |
| Berlin | *n* (%) | 41 (4.16) |  |
| Brandenburg | *n* (%) | 33 (3.35) |  |
| Bremen | *n* (%) | 8 (0.81) |  |
| Hamburg | *n* (%) | 23 (2.33) |  |
| Hesse | *n* (%) | 72 (7.30) |  |
| Mecklenburg-Vorpommern | *n* (%) | 21 (2.13) |  |
| Lower Saxony | *n* (%) | 89 (9.03) |  |
| North Rhine-Westphalia | *n* (%) | 223 (22.62) |  |
| Rhineland-Palatinate | *n* (%) | 45 (4.56) |  |
| Saarland | *n* (%) | 14 (1.42) |  |
| Saxony | *n* (%) | 44 (4.46) |  |
| Saxony-Anhalt | *n* (%) | 31 (3.14) |  |
| Schleswig-Holstein | *n* (%) | 37 (3.75) |  |
| Thuringia | *n* (%) | 25 (2.54) |  |
| **Subjective numeracy** |  |  | *Χ^2^*(6) = 9.996, *p* = 0.12 |
| Low | *n* (%) | 471 (47.77) |  |
| High | *n* (%) | 515 (52.23) |  |
| **Objective graph literacy** |  |  | *Χ^2^*(6) = 7.241, *p* = 0.30 |
| Low | *n* (%) | 434 (44.02) |  |
| High | *n* (%) | 552 (55.98) |  |

*Note.* A one-factorial ANOVA (metric variable level) or *Χ^2^*-test (nominal variable level) was conducted to test for differences between the seven conditions. N/A implies that comparison was not possible (only one group) or reasonable (this is the case for the variable state). ^a^ The response options “Up to 9 years of school education” and “At least 10 years (without university entrance qualification)” were summarized to “Without university entrance qualification” for statistical analysis. ^b^ Participants indicating this response option were excluded from the final sample.

## Supplement S5

**Table S.3**

*Results of a Welch’s t-test with perceived risk of getting vaccinated with AstraZeneca vaccine as dependent variable (Figure 2, Panel A; Study 2; N = 986)*

| Factor | *n* | *M(SD)* | df | *t* | *p*-value | Cohens *d* 95% CI [LL, UL] |
| --- | --- | --- | --- | --- | --- | --- |
| No infographic (control) | 143 | 4.48(1.87) | 195.24 | 2.3861 | 0.02 | 0.34 [0.06, 0.62] |
| Infographic | 843 | 4.08(1.90) |  |  |  |  |

**Table S.4**

*Results of a Welch’s t-test with intention to get vaccinated with AstraZeneca vaccine as dependent variable (Figure 2, Panel C; Study 2; N = 986)*

| Factor | *n* | *M(SD)* | df | *t* | *p*-value | Cohens *d* 95% CI [LL, UL] |
| --- | --- | --- | --- | --- | --- | --- |
| No infographic (control) | 143 | 3.33(2.18) | 195.63 | 0.2762 | 0.78 | 0.04  [-0.24, 0.32] |
| Infographic | 843 | 3.27(2.23) |  |  |  |  |

**Table S.5**

*Results of a 2 x 2 ANOVA with perceived risk of getting vaccinated with AstraZeneca vaccine as dependent variable (Figure 2, Panel D; Study 2; N = 986)*

| Factor | df | Sum square | Mean square | *F* | *p*-value |
| --- | --- | --- | --- | --- | --- |
| Confidence in AstraZeneca (low vs. high) | 1 | 162 | 161.52 | 46.989 | <0.001 |
| Infographic (no vs. yes) | 1 | 19 | 19.20 | 5.586 | 0.02 |
| Confidence*Infographic | 1 | 2 | 2.38 | 0.692 | 0.41 |
| Residuals | 982 | 3375 | 3.44 |  |  |

**Table S.6**

*Results of a 2 x 2 ANOVA with perceived probability of blood clots due to with AstraZeneca as dependent variable (Figure 2, Panel E; Study 2; N = 986)*

| Factor | df | Sum square | Mean square | *F* | *p*-value |
| --- | --- | --- | --- | --- | --- |
| Confidence in AstraZeneca (low vs. high) | 1 | 514.5 | 514.5 | 195.633 | <0.001 |
| Infographic (no vs. yes) | 1 | 0.2 | 0.2 | 0.090 | 0.76 |
| Confidence*Infographic | 1 | 1.5 | 1.5 | 0.584 | 0.45 |
| Residuals | 982 | 2582.8 | 2.6 |  |  |

**Table S.7**

*Results of a 2 x 2 ANOVA with intention to get vaccinated with AstraZeneca vaccine as dependent variable (Figure 2, Panel F; Study 2; N = 986)*

| Factor | df | Sum square | Mean square | *F* | *p*-value |
| --- | --- | --- | --- | --- | --- |
| Confidence in AstraZeneca (low vs. high) | 1 | 1596 | 1595.8 | 481.547 | <0.001 |
| Infographic (no vs. yes) | 1 | 1 | 0.8 | 0.234 | 0.63 |
| Confidence*Infographic | 1 | 1 | 0.8 | 0.256 | 0.61 |
| Residuals | 982 | 3254 | 3.3 |  |  |

**Table S.8**

*Results of a 2 x 2 ANOVA with perceived risk of getting vaccinated with AstraZeneca vaccine as dependent variable (Figure 2, Panel G; Study 2; N = 986)*

| Factor | df | Sum square | Mean square | *F* | *p*-value |
| --- | --- | --- | --- | --- | --- |
| Subjective numeracy (low vs. high) | 1 | 10 | 9.566 | 2.666 | 0.10 |
| Infographic (no vs. yes) | 1 | 19 | 18.953 | 5.282 | 0.02 |
| Subjective numeracy*Infographic | 1 | 7 | 6.577 | 1.833 | 0.18 |
| Residuals | 982 | 3523 | 3.588 |  |  |

**Table S.9**

*Results of a 2 x 2 ANOVA with perceived probability of blood clots due to with AstraZeneca as dependent variable (Figure 2, Panel H; Study 2; N = 986)*

| Factor | df | Sum square | Mean square | *F* | *p*-value |
| --- | --- | --- | --- | --- | --- |
| Subjective numeracy (low vs. high) | 1 | 79.7 | 79.65 | 25.944 | <0.001 |
| Infographic (no vs. yes) | 1 | 0.1 | 0.09 | 0.029 | 0.87 |
| Subjective numeracy*Infographic | 1 | 4.5 | 4.49 | 1.462 | 0.23 |
| Residuals | 982 | 3014.9 | 3.07 |  |  |

*Note.* The assumption of variance homogeneity did not hold (*F*(3,982) = 5.076, *p* < 0.01). Mann-Whitney-U tests confirmed the main effects (Subjective numeracy: *U* = 143166,

*p* <0.001; Infographic: *U* = 61395, *p* = 0.72).

**Table S.10**

*Results of a 2 x 2 ANOVA with intention to get vaccinated with AstraZeneca vaccine as dependent variable (Figure 2, Panel I; Study 2; N = 986)*

| Factor | df | Sum square | Mean square | *F* | *p*-value |
| --- | --- | --- | --- | --- | --- |
| Subjective numeracy (low vs. high) | 1 | 97 | 97.4 | 20.132 | <0.001 |
| Infographic (no vs. yes) | 1 | 1 | 0.97 | 0.201 | 0.65 |
| Subjective numeracy*Infographic | 1 | 2 | 2.04 | 0.421 | 0.52 |
| Residuals | 982 | 4751 | 4.84 |  |  |

*Note.* The assumption of variance homogeneity did not hold (*F*(3,982) = 7.793, *p* < 0.001). Mann-Whitney-U tests confirmed the main effects (Subjective numeracy: *U* = 102740, *p* <0.001; Infographic: *U* = 61204, *p* = 0.76).

## Supplement S6

**Table S.11**

*Results of a Welch’s t-test with perceived probability of blood clotting due to the AstraZeneca vaccine as dependent variable when excluding participants who failed the comprehension check on the first attempt (N = 785)*

| Factor | *n* | *M(SD)* | df | *t* | *p*-value | Cohens *d* 95% CI [LL, UL] |
| --- | --- | --- | --- | --- | --- | --- |
| Control | 113 | 3.77 1.81 | 150.64 | 0.2466 | 0.81 | 0.04 |
| Infographics | 672 | 3.72 1.78 |  |  |  | [-0.28, 0.36] |

**Table S.12**

*Results of a 2 x 2 ANOVA with perceived probability of blood clotting due to the AstraZeneca vaccine as dependent variable when excluding participants who failed the comprehension check on the first attempt (N = 785)*

| Predictor | Sum of squares | *df* | Mean square | *F* | *p* | $\eta_{p}^{2}$ |
| --- | --- | --- | --- | --- | --- | --- |
| Exposure risk (low vs. high) |  |  |  |  |  |  |
| Infographic design (Numbers vs. Circle vs. Manikin) | 1.30 | 1 | 1.29 | 0.41 | 0.52 | < .01 |
| Helmert contrast | 3.20 | 2 | 1.60 | 0.51 | 0.60 | < .01 |
| Contrast 1: Numbers vs. Icons | 3.10 | 1 | 3.12 | 0.99 | 0.32 |  |
| Contrast 2: Circle vs. Manikin | 0.10 | 1 | 0.08 | 0.03 | 0.87 |  |
| Exposure risk* Infographic design | 3.50 | 2 | 1.76 | 0.55 | 0.58 | < .01 |
| Exposure risk* Contrast 1 | 0.90 | 1 | 0.86 | 0.27 | 0.60 |  |
| Exposure risk* Contrast 2 | 2.70 | 1 | 2.65 | 0.84 | 0.36 |  |
| **Residuals** | 2108.10 | 666 | 3.27 |  |  |  |

**Table S.13**

*Results of a 2 x 3 x 2 ANOVA with perceived probability of blood clotting due to the AstraZeneca vaccine as dependent variable and subjective numeracy as explorative factor when excluding participants who failed the comprehension check on the first attempt (N = 785)*

| Predictor | Sum of squares | *df* | Mean square | *F* | *p* | $\eta_{p}^{2}$ |
| --- | --- | --- | --- | --- | --- | --- |
| Exposure risk (low vs. high) | 1.30 | 1 | 1.29 | 0.42 | 0.52 | < .01 |
| Infographic design (Numbers vs. Circle vs. Manikin) | 3.20 | 2 | 1.60 | 0.52 | 0.59 | < .01 |
| Subjective Numeracy (low vs. high) | 70.60 | 1 | 70.58 | 23.07 | < 0.001 | .03 |
| Exposure risk* Infographic design | 3.20 | 2 | 1.58 | 0.52 | 0.60 | < .01 |
| Infographic design* Subjective numeracy | 10.80 | 1 | 10.79 | 3.53 | 0.06 | < .01 |
| Exposure risk* Infographic design* Subjective numeracy | 1.80 | 2 | 0.89 | 0.29 | 0.75 | < .01 |
| Exposure risk* Infographic design* Subjective numeracy | 5.90 | 2 | 2.96 | 0.97 | 0.38 | < .01 |
| **Residuals** | 2019.40 | 660 | 3.06 |  |  |  |

**Table S.13**

*Results of a 2 x 3 x 2 ANOVA with perceived probability of blood clotting due to the AstraZeneca vaccine as dependent variable and graph literacy as explorative factor when excluding participants who failed the comprehension check on the first attempt (N = 785)*

| Predictor | Sum of squares | *df* | Mean square | *F* | *p* | $\eta_{p}^{2}$ |
| --- | --- | --- | --- | --- | --- | --- |
| Exposure risk (low vs. high) | 1.30 | 1 | 1.29 | 0.41 | 0.52 | < .01 |
| Infographic design (Numbers vs. Circle vs. Manikin) | 3.20 | 2 | 1.60 | 0.51 | 0.60 | < .01 |
| Graph literacy (low vs. high) | 14.20 | 1 | 14.25 | 4.51 | 0.03 | < .01 |
| Exposure risk* Infographic design | 2.70 | 2 | 1.37 | 0.43 | 0.65 | < .01 |
| Infographic design* Graph literacy | 0.30 | 1 | 0.32 | 0.10 | 0.75 | < .01 |
| Exposure risk* Infographic design* Graph literacy | 7.10 | 2 | 3.55 | 1.13 | 0.33 | < .01 |
| Exposure risk* Infographic design* Graph literacy | 3.70 | 2 | 1.86 | 0.59 | 0.56 | < .01 |
| **Residuals** | 2083.50 | 660 | 3.16 |  |  |  |

**Table S.14**

*Linear regression for intention to get vaccinated with the AstraZeneca vaccine (Study 2) when excluding participants who failed the comprehension check on the first attempt (N = 785)*

|  | **DV: Intention to get vaccinated with the AstraZeneca vaccine** | | | | | | | | | |  |
| --- | --- | --- | --- | --- | --- | --- | --- | --- | --- | --- | --- |
|  | *Step 1* | | | | |  | *Step 2* | | | | |
| *Predictors* | *B* | 95% CI for *B* | *ß* | 95% CI for *ß* | *p* |  | *B* | 95% CI for *B* | *ß* | 95% CI for *ß* | *p* |
| (Intercept) | 6.07 | 5.58 – 6.65 | -0.00 | -0.05 – 0.05 | **< 0.001** |  | 3.33 | 2.57 – 4.09 | -0.01 | -0.10 – 0.07 | **< 0.001** |
| Perceived probability of becoming infected with COVID-19 | 0.14 | 0.04 – 0.23 | 0.09 | 0.03 – 0.15 | **< 0.01** |  | 0.12 | 0.05 – 0.20 | 0.08 | 0.03 – 0.13 | **0.002** |
| Perceived severity of becoming infected with COVID-19 | 0.28 | 0.19 – 0.37 | 0.20 | 0.13 – 0.26 | **< 0.001** |  | 0.10 | 0.03 – 0.18 | 0.07 | 0.02 – 0.13 | **0.007** |
| Perceived probability of blood clots due to the AZ vaccine | -0.59 | -0.66 – -0.51 | -0.46 | -0.52 – -0.40 | **< 0.001** |  | -0.24 | -0.31 – -0.17 | -0.19 | -0.24 – -0.13 | **< 0.001** |
| Perceived severity of blood clots due to the AZ vaccine | -0.40 | -0.50 – -0.31 | -0.25 | -0.31 – -0.19 | **< 0.001** |  | -0.16 | -0.24 – -0.08 | -0.10 | -0.15 – -0.05 | **< 0.001** |
| Age (years) |  |  |  |  |  |  | 0.02 | 0.01 – 0.02 | 0.11 | 0.06 – 0.15 | **< 0.001** |
| Gender (reference category: female) |  |  |  |  |  |  | -0.23 | -0.42 – -0.03 | -0.10 | -0.19 – -0.01 | **0.026** |
| UEQ (reference category: no UEQ) |  |  |  |  |  |  | 0.26 | 0.06 – 0.47 | 0.12 | 0.03 – 0.21 | **0.012** |
| Confidence in AZ |  |  |  |  |  |  | 0.48 | 0.42 – 0.55 | 0.42 | 0.26 – 0.48 | **< 0.001** |
| Calculation regarding AZ |  |  |  |  |  |  | -0.02 | -0.08 – 0.05 | -0.01 | -0.06 – 0.03 | 0.61 |
| Preference of alternatives to AZ |  |  |  |  |  |  | -0.31 | -0.39 – -0.23 | -0.22 | -0.28 – -0.16 | **< 0.001** |
| N | 785 |  |  |  |  |  | 785 | | | | |
| R^2^ / R^2^ adjusted | 0.423 / 0.420 | | | | |  | 0.638 / 0.633 | | | | |

## Supplement S7

**Table S.15**

*Results of a Welch’s t-test with perceived probability of becoming infected with the coronavirus as dependent variable and condition of Experiment 2 by Schmid & Betsch (2022) as group variable (N = 986)*

| Factor | *n* | *M(SD)* | df | *t* | *p*-value | Cohens *d* 95% CI [LL, UL] |
| --- | --- | --- | --- | --- | --- | --- |
| Control | 487 | 3.60(1.52) | 983.99 | -0.0987 | 0.92 | -0.00 |
| Prebunking | 499 | 3.61(1.55) |  |  |  | [-0.13, 0.12] |

**Table S.16**

*Results of a Welch’s t-test with perceived severity* *of an infection with the coronavirus and condition of Experiment 2 by Schmid & Betsch (2022) as group variable (N = 986)*

| Factor | *n* | *M(SD)* | df | *t* | *p*-value | Cohens *d* 95% CI [LL, UL] |
| --- | --- | --- | --- | --- | --- | --- |
| Control | 487 | 4.02(1.52) | 979.77 | -0.0336 | 0.97 | -0.00 |
| Prebunking | 499 | 4.03(1.67) |  |  |  | [-0.13, 0.12] |

**Table S.17**

*Results of a Welch’s t-test with perceived probability of blood clotting due to the AstraZeneca vaccine as dependent variable and condition of Experiment 2 by Schmid & Betsch (2022) as group variable (N = 986)*

| Factor | *n* | *M(SD)* | df | *t* | *p*-value | Cohens *d* 95% CI [LL, UL] |
| --- | --- | --- | --- | --- | --- | --- |
| Control | 487 | 3.78(1.78) | 983.37 | -0.6496 | 0.52 | -0.04 |
| Prebunking | 499 | 3.85(1.77) |  |  |  | [-0.17, 0.08] |

**Table S.18**

*Results of a Welch’s t-test with perceived severity of blood clotting due to the AstraZeneca vaccine as dependent variable and condition of Experiment 2 by Schmid & Betsch (2022) as group variable (N = 986)*

| Factor | *n* | *M(SD)* | df | *t* | *p*-value | Cohens *d* 95% CI [LL, UL] |
| --- | --- | --- | --- | --- | --- | --- |
| Control | 487 | 5.46(1.45) | 982.58 | 0.4525 | 0.65 | 0.03 |
| Prebunking | 499 | 5.42(1.54) |  |  |  | [-0.10, 0.15] |

**Table S.19**

*Results of a Welch’s t-test with perceived risk of getting vaccinated with AstraZeneca vaccine as dependent variable and condition of Experiment 2 by Schmid & Betsch (2022) as group variable (N = 986)*

| Factor | *n* | *M(SD)* | df | *t* | *p*-value | Cohens *d* 95% CI [LL, UL] |
| --- | --- | --- | --- | --- | --- | --- |
| Control | 487 | 4.09(1.90) | 983.21 | -0.6928 | 0.49 | -0.04 |
| Prebunking | 499 | 4.18(1.90) |  |  |  | [-0.17 – 0.08] |

**Table S.20**

*Results of a Welch’s t-test with intention to get vaccinated with AstraZeneca vaccine as dependent variable and condition of Experiment 2 by Schmid & Betsch (2022) as group variable (N = 986)*

| Factor | *n* | *M(SD)* | df | *t* | *p*-value | Cohens *d* 95% CI [LL, UL] |
| --- | --- | --- | --- | --- | --- | --- |
| Control | 487 | 3.24(2.20) | 983.98 | -0.6402 | 0.52 | -0.04 |
| Prebunking | 499 | 3.33(2.24) |  |  |  | [-0.17 – 0.08] |
